# 3-Methyl­sulfanyl-5-phenyl-4*H*-1,2,4-triazol-4-amine–water (6/1)

**DOI:** 10.1107/S1600536808041056

**Published:** 2009-03-06

**Authors:** Deng-Ze Wu, Miao-Chang Liu, Hua-Yue Wu, Xiao-Bo Huang, Jian-Jun Li

**Affiliations:** a School of Chemistry and Materials Science, Wenzhou University, Zhejiang Wenzhou 325027, People’s Republic of China; b Zhejiang Key Laboratory of Pharmaceutical Engineering, College of Pharmaceutical Sciences, Zhejiang University of Technology, Zhejiang Hangzhou 310014, People’s Republic of China

## Abstract

In the title compound, 6C_9_H_10_N_4_S·H_2_O, the dihedral angle between the five-membered triazole ring and the phenyl ring is 44.33 (16)°. The solvent water molecule is disordered about a special position with 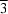 symmetry and its occupancy cannot be greater than 0.1667. The crystal structure is stabilized by inter­molecular N—H⋯N and C–H⋯N hydrogen bonds.

## Related literature

For general background to 1,2,4-triazoles, see: Feng *et al.* (1992 ▶); Hui *et al.* (2000 ▶); Prasad *et al.* (1989 ▶); Mohan *et al.* (1987 ▶) For related structures, see: Xiang *et al.* (2004 ▶); Jin *et al.* (2004 ▶)
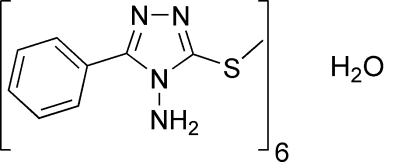


## Experimental

### 

#### Crystal data

6C_9_H_10_N_4_S·H_2_O
*M* *_r_* = 1255.73
Hexagonal, 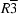

*a* = 23.0266 (15) Å
*c* = 10.5190 (9) Å
*V* = 4830.2 (6) Å^3^
*Z* = 3
Mo *K*α radiation
μ = 0.27 mm^−1^
*T* = 298 K
0.32 × 0.23 × 0.15 mm

#### Data collection

Bruker SMART CCD area-detector diffractometer
Absorption correction: multi-scan (*SADABS*; Bruker, 2002 ▶) *T*_min_ = 0.918, *T*_max_ = 0.960
8946 measured reflections
2011 independent reflections
1647 reflections with *I* > 2σ(*I*)
*R*_int_ = 0.047

#### Refinement

*R*[*F* ^2^ > 2σ(*F* ^2^)] = 0.073
*wR*(*F* ^2^) = 0.204
*S* = 0.99
2011 reflections
137 parameters
H-atom parameters constrained
Δρ_max_ = 0.70 e Å^−3^
Δρ_min_ = −0.27 e Å^−3^

### 

Data collection: *SMART* (Bruker, 2002 ▶); cell refinement: *SAINT* (Bruker, 2002 ▶); data reduction: *SAINT*; program(s) used to solve structure: *SHELXS97* (Sheldrick, 2008 ▶); program(s) used to refine structure: *SHELXL97* (Sheldrick, 2008 ▶); molecular graphics: *SHELXTL* (Sheldrick, 2008 ▶); software used to prepare material for publication: *SHELXTL*.

## Supplementary Material

Crystal structure: contains datablocks I, global. DOI: 10.1107/S1600536808041056/sj2552sup1.cif

Structure factors: contains datablocks I. DOI: 10.1107/S1600536808041056/sj2552Isup2.hkl

Additional supplementary materials:  crystallographic information; 3D view; checkCIF report

## Figures and Tables

**Table 1:** Hydrogen-bond geometry (Å, °)

*D*—H⋯*A*	*D*—H	H⋯*A*	*D*⋯*A*	*D*—H⋯*A*
N4—H4*A*⋯N1^i^	0.86	2.30	3.127 (4)	162
N4—H4*B*⋯N2^ii^	0.86	2.21	3.060 (4)	172
C5—H5⋯N1^i^	0.93	2.60	3.507 (6)	167

Symmetry codes: (i) 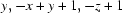; (ii) 
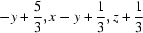
.

**Fig. 1.**
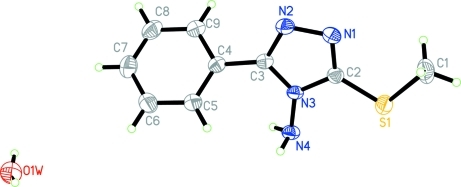
The formula unit of (I) with atom numbering, showing displacement ellipsoids at the 50% probability level.

**Fig. 2.**
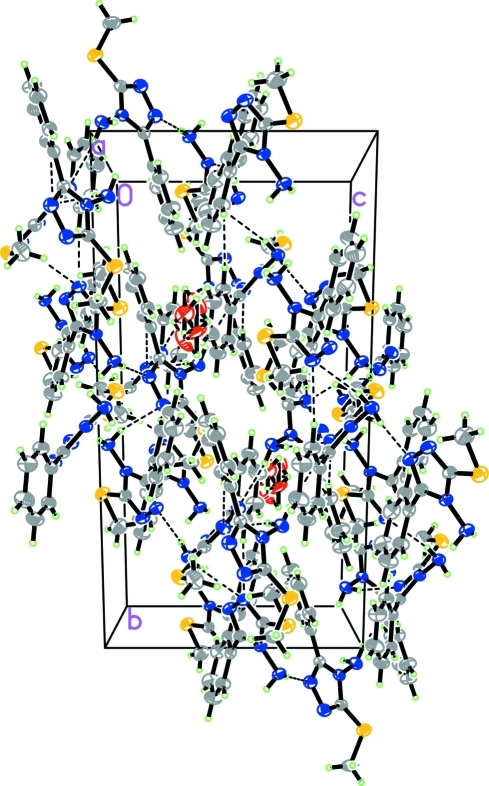
Part of the crystal structure, showing the infinite hydrogen-bonding network of (I) running along the a axis. Hydrogen bonds are indicated by dashed lines.

## supplementary crystallographic information

### Comment

A literature survey reveals that 1,2,4-triazoles are good intermediates in the synthesis of some fused heterocycles which exhibit various biological properties, including antimicrobial (Feng *et al.*, 1992), antibacterial and antifungal (Hui *et al.*, 2000), anti-inflammatory (Prasad *et al.*, 1989) and diuretic (Mohan & Anjaneyulu, 1987) activities.

The molecule of (I), Fig. 1, contains a five-membered triazole ring A(N1,N2,C3,N3,C2) with a benzene ring substituent B(C1—C6). The two rings are each essentially planar, with average deviations from planarity of 0.003 (1) and 0.004 (1) Å, respectively. The dihedral angle between the thiadiazole ring and the benzene ring is 44.33 (16)°.

The water molecule is disordered about a threefold inversion axis such that the asymmetric unit comprises one C_9_H_10_N_4_S molecule and a water molecule with occupancy *ca* 0.167

The C—N bond lengths in the molecule lie in the range 1.302 (5)–1.364 (4) Å. These are longer than a typical double C=N bond [*ca* 1.269 (2) Å] (Xiang *et al.*, 2004), but shorter than a C—N single bond [*ca* 1.443 (4) Å] (Jin *et al.*, 2004), indicating a degree of electron delocalization in the triazole ring.

The crystal packing in (I), Fig. 2, is stabilized by intermolecular and intramolecular N—H···N and C–H···N hydrogen bonds, Table 1.

### Experimental

4-Amino-5-phenyl-2,4-dihydro[1,2,4]triazole-3-thione(0.96 g 5.0 mmol), methyl iodide(1.07 g 7.5 mmol) and sodium hydroxide(0.28 g 7.0 mmol) were dissolved in stirred dichloromethane (30 ml)and left for 2 h at room temperature. The resulting solid was filtered off and recrystallized from ethanol to give the title compound in 73% yield. Crystals suitable for X-ray analysis were obtained by slow evaporation of a ethanol solution at room temperature (m.p. 425–426 K).

### Refinement

H atoms bound to N and O atoms were found in difference Fourier maps and their distances restrainted to N—H = 0.86 (2)Å and O—H = 0.85 (2)Å with *U*_iso_ = 1.2*U*_eq_ (parent atom), respectively. All other H atoms were positioned geometrically and allowed to ride on their parent atoms at distances of C*sp*^2^—H = 0.93 Å with *U*_iso_ = 1.2 *U*_eq_ (parent atom), *C*(methyl)-H = 0.96 Å with *U*_iso_ = 1.5 *U*_eq_ (parent atom).

### Crystal data

**Table:**

6C_9_H_10_N_4_S·H_2_O	*D*_x_ = 1.314 Mg m^−^^3^
*M**_r_* = 1255.73	Mo *K*α radiation, λ = 0.71073 Å
Hexagonal, *R*3	Cell parameters from 2375 reflections
Hall symbol: -R 3	θ = 2.2–23.0°
*a* = 23.0266 (15) Å	µ = 0.27 mm^−^^1^
*c* = 10.5190 (9) Å	*T* = 298 K
*V* = 4830.2 (6) Å^3^	Prism, colorless
*Z* = 3	0.32 × 0.23 × 0.15 mm
*F*(000) = 2004	

### Data collection

**Table:**

Bruker SMART CCD area-detector diffractometer	2011 independent reflections
Radiation source: fine-focus sealed tube	1647 reflections with *I* > 2σ(*I*)
graphite	*R*_int_ = 0.047
φ and ω scans	θ_max_ = 25.5°, θ_min_ = 1.8°
Absorption correction: multi-scan (*SADABS*; Bruker, 2002)	*h* = −27→27
*T*_min_ = 0.918, *T*_max_ = 0.960	*k* = −27→20
8946 measured reflections	*l* = −10→12

### Refinement

**Table:**

Refinement on *F*^2^	Primary atom site location: structure-invariant direct methods
Least-squares matrix: full	Secondary atom site location: difference Fourier map
*R*[*F*^2^ > 2σ(*F*^2^)] = 0.073	Hydrogen site location: inferred from neighbouring sites
*wR*(*F*^2^) = 0.204	H-atom parameters constrained
*S* = 0.99	*w* = 1/[σ^2^(*F*_o_^2^) + (0.1049*P*)^2^ + 19.9182*P*] where *P* = (*F*_o_^2^ + 2*F*_c_^2^)/3
2011 reflections	(Δ/σ)_max_ < 0.001
137 parameters	Δρ_max_ = 0.70 e Å^−^^3^
0 restraints	Δρ_min_ = −0.27 e Å^−^^3^

### Special details

**Table:**

Geometry. All esds (except the esd in the dihedral angle between two l.s. planes) are estimated using the full covariance matrix. The cell esds are taken into account individually in the estimation of esds in distances, angles and torsion angles; correlations between esds in cell parameters are only used when they are defined by crystal symmetry. An approximate (isotropic) treatment of cell esds is used for estimating esds involving l.s. planes.
Refinement. Refinement of F^2^ against ALL reflections. The weighted R-factor wR and goodness of fit S are based on F^2^, conventional R-factors R are based on F, with F set to zero for negative F^2^. The threshold expression of F^2^ > 2sigma(F^2^) is used only for calculating R-factors(gt) etc. and is not relevant to the choice of reflections for refinement. R-factors based on F^2^ are statistically about twice as large as those based on F, and R- factors based on ALL data will be even larger.

### Fractional atomic coordinates and isotropic or equivalent isotropic displacement parameters (Å^2^)

**Table:**

	*x*	*y*	*z*	*U*_iso_*/*U*_eq_	Occ. (<1)
S1	1.03285 (6)	0.89151 (5)	0.71331 (11)	0.0608 (4)	
O1W	0.645 (2)	0.348 (3)	0.356 (2)	0.095 (12)	0.166667
H1W	0.6667	0.3333	0.3967	0.142*	0.50
H2W	0.6439	0.3779	0.3102	0.142*	0.166667
N1	1.08197 (15)	0.83742 (16)	0.5409 (3)	0.0520 (8)	
N2	1.05881 (15)	0.77523 (16)	0.4828 (3)	0.0501 (8)	
N3	0.98055 (13)	0.76822 (14)	0.6086 (3)	0.0381 (7)	
N4	0.92266 (14)	0.74747 (15)	0.6827 (3)	0.0457 (8)	
H4A	0.8918	0.7438	0.6318	0.055*	
H4B	0.9136	0.7090	0.7124	0.055*	
C1	1.1062 (3)	0.9644 (2)	0.6516 (5)	0.0809 (15)	
H1A	1.1442	0.9579	0.6578	0.121*	
H1B	1.1146	1.0031	0.7001	0.121*	
H1C	1.0989	0.9709	0.5642	0.121*	
C2	1.03399 (18)	0.83096 (18)	0.6157 (3)	0.0433 (9)	
C3	0.99830 (17)	0.73476 (18)	0.5243 (3)	0.0410 (8)	
C4	0.95704 (18)	0.66410 (18)	0.4868 (3)	0.0427 (8)	
C5	0.8903 (2)	0.6366 (2)	0.4557 (4)	0.0555 (10)	
H5	0.8695	0.6622	0.4626	0.067*	
C6	0.8543 (2)	0.5711 (2)	0.4142 (5)	0.0726 (13)	
H6	0.8092	0.5526	0.3935	0.087*	
C7	0.8845 (3)	0.5333 (2)	0.4034 (5)	0.0783 (14)	
H7	0.8600	0.4892	0.3746	0.094*	
C8	0.9510 (3)	0.5600 (2)	0.4349 (5)	0.0689 (13)	
H8	0.9714	0.5341	0.4276	0.083*	
C9	0.9874 (2)	0.6252 (2)	0.4773 (4)	0.0524 (10)	
H9	1.0322	0.6431	0.4996	0.063*	

### Atomic displacement parameters (Å^2^)

**Table:**

	*U*^11^	*U*^22^	*U*^33^	*U*^12^	*U*^13^	*U*^23^
S1	0.0603 (7)	0.0493 (6)	0.0676 (7)	0.0236 (5)	0.0099 (5)	−0.0071 (5)
O1W	0.050 (15)	0.09 (3)	0.100 (18)	0.001 (10)	0.041 (12)	−0.015 (14)
N1	0.0389 (17)	0.0496 (19)	0.060 (2)	0.0166 (15)	0.0097 (15)	0.0029 (15)
N2	0.0428 (18)	0.0512 (19)	0.0565 (19)	0.0236 (15)	0.0112 (14)	0.0018 (15)
N3	0.0322 (14)	0.0406 (16)	0.0436 (16)	0.0197 (13)	0.0060 (12)	0.0054 (12)
N4	0.0378 (16)	0.0474 (17)	0.0534 (19)	0.0225 (14)	0.0097 (13)	0.0090 (14)
C1	0.079 (3)	0.044 (2)	0.095 (4)	0.012 (2)	0.013 (3)	−0.005 (2)
C2	0.0404 (19)	0.0412 (19)	0.048 (2)	0.0203 (16)	0.0030 (16)	0.0040 (15)
C3	0.0404 (19)	0.045 (2)	0.0417 (19)	0.0245 (16)	0.0027 (15)	0.0037 (15)
C4	0.048 (2)	0.045 (2)	0.0392 (19)	0.0258 (17)	0.0067 (15)	0.0056 (15)
C5	0.050 (2)	0.053 (2)	0.069 (3)	0.029 (2)	−0.0036 (19)	−0.005 (2)
C6	0.056 (3)	0.057 (3)	0.100 (4)	0.025 (2)	−0.012 (2)	−0.012 (3)
C7	0.080 (3)	0.048 (3)	0.102 (4)	0.028 (2)	0.000 (3)	−0.014 (2)
C8	0.081 (3)	0.060 (3)	0.081 (3)	0.047 (3)	0.010 (3)	0.001 (2)
C9	0.055 (2)	0.056 (2)	0.054 (2)	0.033 (2)	0.0063 (18)	0.0038 (18)

### Geometric parameters (Å, °)

**Table:**

S1—C2	1.743 (4)	N4—H4B	0.8600
S1—C1	1.804 (5)	C1—H1A	0.9600
O1W—O1W^i^	0.88 (3)	C1—H1B	0.9600
O1W—O1W^ii^	0.88 (3)	C1—H1C	0.9600
O1W—O1W^iii^	1.28 (4)	C3—C4	1.470 (5)
O1W—O1W^iv^	1.28 (4)	C4—C5	1.378 (5)
O1W—O1W^v^	1.55 (4)	C4—C9	1.388 (5)
O1W—H1W	0.8501	C5—C6	1.379 (6)
O1W—H2W	0.8500	C5—H5	0.9300
N1—C2	1.302 (5)	C6—C7	1.365 (7)
N1—N2	1.395 (5)	C6—H6	0.9300
N2—C3	1.305 (5)	C7—C8	1.375 (7)
N3—C2	1.352 (5)	C7—H7	0.9300
N3—C3	1.364 (4)	C8—C9	1.377 (6)
N3—N4	1.406 (4)	C8—H8	0.9300
N4—H4A	0.8601	C9—H9	0.9300
			
C2—S1—C1	98.7 (2)	S1—C1—H1C	109.5
O1W^i^—O1W—O1W^ii^	93 (4)	H1A—C1—H1C	109.5
O1W^i^—O1W—O1W^iii^	89.994 (4)	H1B—C1—H1C	109.5
O1W^ii^—O1W—O1W^iv^	89.995 (9)	N1—C2—N3	110.8 (3)
O1W^iii^—O1W—O1W^iv^	60.000 (8)	N1—C2—S1	128.0 (3)
O1W^i^—O1W—O1W^v^	55.4 (9)	N3—C2—S1	121.2 (3)
O1W^ii^—O1W—O1W^v^	55.4 (9)	N2—C3—N3	109.2 (3)
O1W^i^—O1W—H1W	85.0	N2—C3—C4	124.7 (3)
O1W^ii^—O1W—H1W	85.0	N3—C3—C4	126.1 (3)
O1W^v^—O1W—H1W	48.2	C5—C4—C9	119.5 (4)
O1W^i^—O1W—H2W	67.8	C5—C4—C3	121.8 (3)
O1W^ii^—O1W—H2W	108.1	C9—C4—C3	118.6 (3)
O1W^iii^—O1W—H2W	144.8	C4—C5—C6	119.9 (4)
O1W^iv^—O1W—H2W	110.0	C4—C5—H5	120.0
O1W^v^—O1W—H2W	117.3	C6—C5—H5	120.0
H1W—O1W—H2W	149.9	C7—C6—C5	120.4 (4)
C2—N1—N2	106.3 (3)	C7—C6—H6	119.8
C3—N2—N1	108.1 (3)	C5—C6—H6	119.8
C2—N3—C3	105.6 (3)	C6—C7—C8	120.1 (4)
C2—N3—N4	122.3 (3)	C6—C7—H7	119.9
C3—N3—N4	132.0 (3)	C8—C7—H7	119.9
N3—N4—H4A	106.6	C7—C8—C9	120.1 (4)
N3—N4—H4B	104.5	C7—C8—H8	120.0
H4A—N4—H4B	111.3	C9—C8—H8	120.0
S1—C1—H1A	109.5	C8—C9—C4	119.9 (4)
S1—C1—H1B	109.5	C8—C9—H9	120.1
H1A—C1—H1B	109.5	C4—C9—H9	120.1
			
C2—N1—N2—C3	0.4 (4)	N4—N3—C3—C4	1.5 (6)
N2—N1—C2—N3	−0.6 (4)	N2—C3—C4—C5	−135.3 (4)
N2—N1—C2—S1	−179.7 (3)	N3—C3—C4—C5	45.9 (5)
C3—N3—C2—N1	0.6 (4)	N2—C3—C4—C9	41.9 (5)
N4—N3—C2—N1	178.1 (3)	N3—C3—C4—C9	−137.0 (4)
C3—N3—C2—S1	179.7 (2)	C9—C4—C5—C6	−0.7 (6)
N4—N3—C2—S1	−2.7 (5)	C3—C4—C5—C6	176.4 (4)
C1—S1—C2—N1	11.9 (4)	C4—C5—C6—C7	−0.2 (7)
C1—S1—C2—N3	−167.1 (3)	C5—C6—C7—C8	0.6 (8)
N1—N2—C3—N3	−0.1 (4)	C6—C7—C8—C9	−0.1 (8)
N1—N2—C3—C4	−179.1 (3)	C7—C8—C9—C4	−0.8 (7)
C2—N3—C3—N2	−0.3 (4)	C5—C4—C9—C8	1.2 (6)
N4—N3—C3—N2	−177.5 (3)	C3—C4—C9—C8	−176.0 (4)
C2—N3—C3—C4	178.7 (3)		

Symmetry codes: (i) *y*+1/3, −*x*+*y*+2/3, −*z*+2/3; (ii) *x*−*y*+1/3, *x*−1/3, −*z*+2/3; (iii) −*y*+1, *x*−*y*, *z*; (iv) −*x*+*y*+1, −*x*+1, *z*; (v) −*x*+4/3, −*y*+2/3, −*z*+2/3.

### Hydrogen-bond geometry (Å, °)

**Table:**

*D*—H···*A*	*D*—H	H···*A*	*D*···*A*	*D*—H···*A*
N4—H4A···N1^vi^	0.86	2.30	3.127 (4)	162
N4—H4B···N2^vii^	0.86	2.21	3.060 (4)	172
C5—H5···N1^vi^	0.93	2.60	3.507 (6)	167

Symmetry codes: (vi) *y*, −*x*+*y*+1, −*z*+1; (vii) −*y*+5/3, *x*−*y*+1/3, *z*+1/3.
